# From ideas to studies: how to get ideas and sharpen them into research questions

**DOI:** 10.2147/CLEP.S142940

**Published:** 2018-03-06

**Authors:** Jan P Vandenbroucke, Neil Pearce

**Affiliations:** 1 Leiden University Medical Center, Leiden, the Netherlands; 2 Department of Clinical Epidemiology, Aarhus University, Aarhus, Denmark; 3 Department of Medical Statistics and Centre for Global NCDs, London School of Hygiene and Tropical Medicine, London, UK

**Keywords:** study design, writing a paper, research questions

## Abstract

Where do new research questions come from? This is at best only partially taught in courses or textbooks about clinical or epidemiological research. Methods are taught under the assumption that a researcher already knows the research question and knows which methods will fit that question. Similarly, the real complexity of the thought processes that lead to a scientific undertaking is almost never described in published papers. In this paper, we first discuss how to get an idea that is worth researching. We describe sources of new ideas and how to foster a creative attitude by “cultivating your thoughts”. Only a few of these ideas will make it into a study. Next, we describe how to sharpen and focus a research question so that a study becomes feasible and a valid test of the underlying idea. To do this, the idea needs to be “pruned”. Pruning a research question means cutting away anything that is unnecessary, so that only the essence remains. This includes determining both the latent and the stated objectives, specific pruning questions, and the use of specific schemes to structure reasoning. After this, the following steps include preparation of a brief protocol, conduct of a pilot study, and writing a draft of the paper including draft tables. Then you are ready to carry out your research.

## Introduction

How do you get an idea for a study? How do you turn your idea into a testable hypothesis, and turn this into an appropriate and feasible study design? This is usually at best only partially taught in epidemiology courses. Most courses and textbooks assume that you know your research question and the general methods that you will need to answer it. Somehow it is assumed that you can readily translate your idea into a specific framework, such as the PICO framework (Patient, Intervention, Control or Comparison, Outcome)^1^ or the FINER framework (Feasible, Interesting, Novel, Ethical, and Relevant)^2^ or that you can fit it into counterfactual reasoning.^3^ However, before describing your project in one of these frameworks, you first need to have an idea for your study and think about it in general terms: why you might do a study and how you might do a study.

This paper considers the complex process of having ideas, keeping track of them, turning them into studies, trying them out in pilot studies, and writing a draft paper before you finally embark on your study.

The paper is intended for novice researchers in clinical or public health epidemiology. It is not intended to be a comprehensive literature review about creativity, nor a sociology or philosophical treatise about why scientists get particular ideas (and not other ideas). It is based on our personal experience of (a combined) 70+ epidemiologic research-years. We have worked on very different topics, mostly on opposite sides of the globe, yet found that our experiences are quite similar. The fact that these issues are rarely covered in epidemiology courses has provided motivation to reflect on our experience.

## Getting new ideas

So how do you get an idea? How some juxtaposition of neural patterns in our brain suddenly creates a new idea is a process that we are far from understanding. According to Karl Popper, the origin of new ideas does not matter; the only thing of interest is to devise how to test them.^4^ Over the past decades, the literature has been enriched with new ideas about “being creative” in science – as witnessed in the book *Innovation Generation* by Ness.^5^

In the present paper, we will not cover the literature about creativity and discovery in depth, but we will discuss the issues that we consider relevant to epidemiologic research. We will first consider the more general principles.

The real complexity of the thought processes that lead to a scientific undertaking is almost never described in published papers. Immunologist Medawar claimed that in this respect almost all scientific papers may be a fraud – not in the sense that scientists deliberately produce misleading data, but in the sense that the real thought processes that lead to the data and conclusions are not mentioned.^6^ Scientists tell us about their real thought processes in memoirs, inaugural, or valedictory lectures – which is why these are so much more interesting than “standard” papers or presentations.

### What strikes our minds: regularities or anomalies?

All sciences study a particular “object of knowledge” (eg, “matter”, “life”). Ideas come from experience and previous knowledge or facts about this object of knowledge, although this knowledge is always filtered through the perspective of one or more theories.^7^ Epidemiology studies the distribution and determinants of disease in human populations,^8^ and epidemiological ideas arise from observing and thinking about populations.^9^ These could be clinical populations (ie, clinical experience, sometimes involving just a few patients), exposure-based populations (eg, workers exposed to a particular chemical), or general populations (geographically defined or sociologically defined). Whatever the population we are interested in, ideas come from observing either regularities or anomalies.

The observation of regularities (“induction”) is a common origin of new ideas.^4^,^10^–^13^ Philosopher David Hume described “Induction” as: regularly seeing two things happening in succession (like pushing a switch and a light going on) leads to suspicions of causality. As he pointed out, causality can never be proven by the mere observation of “constant conjunctions”, but observing regularities can start our train of thought.^12^

An anomaly (or irregularity) strikes our mind, because it defies our expectations. The regularity that we expected was our “hypothesis” (even if it was not really explicitly formulated); the anomaly is a “refutation”.^4^,^13^ It forces us to think about other explanations, and these lead to new hypotheses that we then try to test. Thus, scientists do not usually start from hypotheses that are nicely formulated “out of the blue”, but instead start from previous knowledge and experience; when they are challenged by anomalies, scientists seek new explanations.^14^

An interesting way to discover anomalies is to enter a new field of research; since you have other background experience than the people already in the field, you see things that they take for granted but that strike you as odd – at the same time, you may also see new explanations for these anomalies. One of the pioneers of clinical epidemiology, Sackett, once wrote that scientists should “retire” from a field as soon as they become “experts”.^15^ When you are too long in a field, you will no longer see the anomalies, and you may even obstruct newcomers with new explanations. Of course, there are differences between scientists: some roam across various fields and others stick to a problem area that they explore with increasing depth – then the increasing depth and the new techniques that one needs for advancing one’s thoughts will be like a “new field”.

### Taxonomies of discovery

Few researchers have listed the different ways in which one can arrive at new ideas, that is, lists of ways of discovery. We will present two of them – which have very different origins but remarkable similarities. Several examples of studies corresponding to items on these two lists are given in Appendix Examples A1–A10.

Sources for new ideas about health care evaluation were described by Crombie and Davies in the chapter “Developing the research question” of their book on *Research in Health Care* that reflects a UK public health experience.^16^

- “Review existing practice […] the current organisation and delivery of health care is not as good as it could be […]”
- “Challenge accepted ideas […] much of health care is based on accepted practice rather than research evidence […]” (Appendix Example A3)
- “Look for conflicting views […] which indicate either that there is not enough evidence, or that some practitioners are misinformed”
- “Investigate geographical variation […] reflecting on the reasons [for geographical variation] can be a fruitful source of research questions […]” (Appendix Example A6)
- “Identify Cinderella topics […] important areas of health care are often overlooked […]”
- “Let loose the imagination […] look for wild or impossible ideas […] free the mind from the constraints of conventional wisdom […].”

A taxonomy for sources of clinical research questions about medical care and clinical problems was proposed by Hulley and Cummings, in the context of clinical research in the US:^2^

- “Build on experience;” your own experience, that of close colleagues with whom you can freely discuss your research ideas, and that of a good mentor, because young researchers might not yet have much experience, “An essential strategy for a young investigator is to apprentice himself to an experienced senior scientist who has the time and interest to work with him regularly.”
- “Be alert to new ideas”
  ○ By harvesting “the medical literature and attending journal clubs, national and international meetings, seeking informal conversations with other scientists and colleagues”
  ○ “A sceptical attitude about prevailing beliefs can stimulate good research questions”
  ○ Be alert to “careful observation of patients, which has historically been one of the major sources of descriptive studies” (Appendix Examples A1 and A2)
  ○ Your experiences in teaching; having to explain something may make you aware of gaps in your knowledge; questions by patients and colleagues may similarly identify things that we do not fully understand or ignore
- “Keep the imagination roaming […]” by a mixture of creativity and tenacity; “put an unresolved question clearly in view and turn on the mental switch that lets the mind run freely toward it”.

A special mention needs to be made about the last categories of both the lists: “Let loose the imagination” and “Keep the imagination roaming”. These are especially important to find innovative solutions. In many situations wherein you cannot do a perfect study and you run a grave danger of potential confounding or bias, it helps to “get deeply immersed”: to understand the problem biologically, clinically, socially, organizationally, and environmentally will help you to think about what is happening, why it is happening, and whether you can find situations in which the potential confounders or biases do not exist or exists in reverse. You should forget formal designs and think out of the box: you will find instances of studies that mutually reinforce each other and may even arrive at formulating new designs or analytic solutions (see Appendix Examples A7–A10).

### Keeping track of your ideas

It is not only important to have good ideas but also important to develop them. Researchers who work in laboratories have the habit of keeping “lab logs”. They write down briefly the results of an experiment, note why they think it went wrong, and how they will perform the next experiment. This permits them to trace how they changed the experiments or even the content and the direction of their research. We should do the same in epidemiologic and clinical research, particularly in the stage of creating new ideas. Such notes about ideas can include not only hypotheses and views or results by others but also drawing directed acyclic graphs (DAGs) (see “Intermezzo: specific schemes to structure reasoning” section) to make the causal structures of ideas clear.

The greatest minds kept track of their thoughts. Charles Darwin’s notebooks document his ideas, his observations, his readings, and new theories and facts that struck him.^17^ For example, Darwin noted a story that he heard from his father, a medical practitioner. His father recounted that he had been struck by one of his patients’ ways of expressing himself, because he had attended a parent of the patient who had had the same mannerisms – even though the parent had died when the patient was still an infant. Remarks like these still have relevance today when we think about the heredity and evolution of behavior.

The sociologist C Wright Mills carried the description of the process one step further in the appendix of his book on *The Sociological Imagination*.^18^ He encourages young sociologists to set up a file of stacked cards to keep track of “[…] personal experience and professional activities, studies underway and studies planned […]” which “[…] encourages you to capture ‘fringe thoughts’: various ideas which may be by-products of everyday life, stretches of conversations […]”. These notes are continuously reshuffled, regrouped under new headings, and pondered. Mills denounced the habit of most (social) scientists who feel the need to write about their plans only when they are going to apply for a grant. He thought that scientists should continually work with their file of ideas and regularly take stock of how these have evolved.

Such strategies are still relevant today, even if our “logs” are kept in electronic form, particularly because grant writing has become more demanding, hectic, and time-consuming. From such files, new research projects are born: while your ideas gradually develop, you keep wondering what data you might need to prove a certain proposition, and how you might get those data in the easiest way possible. Often, ideas are reshuffled and regrouped under new headings. A new observation, a new piece of literature may make old ones fall into place, or there may suddenly be a new opportunity to work out an old idea.

A complementary advice recently came in a blog from a contemporary sociologist, Aldrich: his advice is to “Write as if you don’t have the data”, that is, to write “[…] the literature review and planning phase of a project, preferably before it has been locked into a specific research design”.^19^

### The role of emotions

Underlying the discovery process, there are often two emotions: “surprise” and “indignation”. Surprise is the intellectual emotion when we see something happening against expectation: a patient with an unusual exposure, unusual disease manifestation, sudden cure, or sudden ill-understood deterioration; a laboratory result that is an anomaly; and a sudden epidemic of disease in a population. Indignation is the moral emotion: a group of patients is not being treated well because we lack sufficient knowledge, or because we are blundering in organizing health care or in transmitting and applying public health knowledge. Some passion is useful to bring any undertaking to a good end, be it that the passion should be restrained and channeled into polite undertakings, like in a research protocol. While doing the research project, maintaining some of the original passion will help you to find ways to overcome the daily hassles of research, the misadventures, the difficulties of getting others to collaborate, and the difficulties of getting published (Appendix Example A11).

## Sharpening the research question: the pruning

Pruning a research question means cutting away anything that is unnecessary, so that only the essence remains.

The initial spark of an idea will usually lead to some rather general research question. Invariably, this is too ambitious, or so all-encompassing that it cannot be researched (at least not within the time frame of a single grant or PhD project). You have to refine your research question into something that is interesting, yet feasible. To do so, you have to know clearly where you are heading. The emphasis on a clear preconceived idea about what you want to attain by your research often comes as a surprise; some people object: “[…] isn’t research about discovery? How can you know in advance what you want to find?”

The social scientist Verschuren proposed the “wristwatch metaphor”.^20^ A researcher is not like a beachcomber, who strolls along the beach to see whether anything valuable washed ashore. Rather, a researcher is like someone who has lost her wristwatch on the beach and returns to search for it. She knows what part of the beach to look, she can describe her wristwatch in detail, and once she has found it, she knows that this is the watch she was looking for. Some further background to these ideas can be found in Appendix B.

Charles Medawar wrote in his *Advice to a Young Scientist* (page 18)^21^ that as much as politics is the ‘art of the possible’, research is the ‘art of the soluble’. A research question should be limited to a question that can be solved with the resources at hand. This does not mean that you should preferentially study “trivial” questions with easy solutions. It does mean that you should seek out your particular niche: something specific, something that was overlooked by others, or some new twist to a general question, so that you can make your own contribution.

The concept of “serendipity” is often invoked when thinking of “seeking novelty”: it means finding something that you were not looking for. For a full discussion of the more complex reality that shows how, in reality, “chance favors a prepared mind”, see Appendix C.

### Proceed in the inverse order of the paper that you will write

From the aforementioned, we know that we need a precise aim and a soluble research question.

How can we achieve this? The best approach is to “begin at the end”, that is, the conclusion that you hope to support when you eventually publish your research findings, perhaps many years from now.^22^ Most medical research papers have a fixed format: introduction, methods, results, discussion. Usually, the discussion has three parts: summary of the results, discussion of the strengths and limitations, and the importance and interpretation of the findings. There you start: you try to imagine what such last lines of the eventual paper might be – in particular what their intent, their message to the reader might be. Another useful strategy would be to imagine what might be written in the separate box “What this paper adds” that many journals nowadays ask to convey the message from the authors clearly and succinctly to the readers.

### The “latent” versus the “stated” objective

The pioneer clinical epidemiologist Feinstein wrote that a good research consultant should be like a good clinician, who first wants to learn from the patient: “What is the chief complaint?”, that is, which is the problem that you want to study. Next, “What will you do with the answer?”^22^ The latter question is not just about the potential conclusions of the research paper, but more importantly, their meaning. What is the intended effect (or impact) of the findings? He called this the “latent objective”: what do you want to achieve or change by your project; the “stated objective” is different, it is the type of result that the study will deliver. For example, the stated objective can be that you want to do a randomized trial to compare one intervention versus another and that you will look at recurrence of disease. The latent objective might be that you are concerned that one intervention may be harmful to patients, driven by special interests, and that if this is the case it should be abolished.

Rather analogously, the long-time editor of the *Annals of Internal Medicine*, Edward Huth, proposed in his book about medical publishing the “So-What” and the “Who-Cares” tests: “What may happen if the paper’s message is correct?”; may it change concepts and treatment or stimulate further exciting research?^23^ In fact, many funders now require such an “impact statement” as part of the grant application process.

Experienced research consultants know that when trying to discover the latent objective, it is useful to brush aside the detailed protocol and to ask directly what the meaning of the research is. The meaning of the research is often not clearly stated in a formal study protocol that limits itself more or less to “stated aims”.^24^ Like a patient who cannot articulate her/his complaints very well, would-be researchers lose themselves in trivial “side issues” or operational details of the protocol. Appendix Examples A2 and A11 explain the importance of elucidating the underlying frustration of the clinician-researcher to clearly guide a research effort.

After initial questions have set the scene and clarified the “latent objective” of a project, the next questions are more operational, translating the latent objective back into a “stated objective”.^22^ The stated objective should be a feasible research project. According to Feinstein, one should ask: what maneuver is to be executed (what intervention, deliberate or not, and how is it administered), what groups are to be compared (and why those groups), and what is the outcome that we will study?

In these phases of discussion, one needs to immerse oneself into the problem: one has to understand it biologically and clinically, and how it is dealt with in the daily practice of health care in the setting in which you will do research. Getting deeply immersed in the problem is the only way of arriving at shrewd or new solutions for studies on vexing medical or public health problems (Appendix Example A9). Mere discussion of technical or procedural aspects of a proposed design, data collection, or analysis will usually not lead to new insights.

### Specific pruning questions, to ask yourself or others

In initial discussions, one goes back and forth between the general aim (the latent objective), the scientific questions that follow from it, and the possible research designs (with stated objectives). After feeling secure about the “latent” aim, proceed with more specific questions.

- Try to describe exactly the knowledge gap that you want to fill (ie, the watch that you lost at the beach). Is it about etiology, about pathogenesis, about prognosis? What should change for the benefit of a particular group of patients? Try to be as specific as possible. Do your colleagues see these problems and their solutions as you do? – and if not, why don’t they?
- Once you know the point you want to make, describe what table or figure you need to fill the gap in knowledge, that is, what would your results look like? This means drawing a simple table or graph. Are these the data you want? Will these tables convince your colleagues? What objections might they have? Keep in mind that if the research results go against ingrained beliefs, they will be scrutinized mercilessly, so the important aspects of your research should be able to withstand likely objections.
- Thereafter, the questions become more practical: what study design is needed to produce this table, this figure? Can we do this? Do we have the resources or can we find them?

### Be self-critical

You should always remain self-critical about the aspects that threaten the validity of your study (Appendix Example A12).^25^ If the practical problems are too large, or the research question too unfeasibly grandiose, it might be wise to settle for a less ambitious aim (Appendix Example A13).

Paraphrasing Miettinen,^26^ the first decision is whether you should do the study at all. There might be several reasons to decide not to pursue a study. One might be that arriving at a satisfactory design will be impossible, because of biases that you are unable to solve. It serves no purpose to add another study that suffers from the same unsolved problems as previous studies. For example, it does not serve any purpose to do yet another study that shows lower mortality in vegetarians, if you cannot solve the problems of confounding that vegetarians are persons who have different lifestyles in comparison with others.^27^ (If, however, you have found a solution – pursue it at all means!) Nevertheless, thinking about the potential problems and ultimate aims of a seemingly impossible question can foster the development of a new study design or a new method of analysis, (Appendix Examples A2, A9, and A10). In the same vein, deciding that you cannot do a study yourself might make you look for collaboration with persons who have the type of data that you do not, for example, in a different population where it is believed that confounding is not so severe or may even be in the opposite direction.

All studies have imperfections, but you need to be aware which ones you can tolerate.^28^ In the early stages of an enquiry, an “imperfect” study might still be worthwhile to see whether “there might be something in it”. For example, time trends or ecological comparisons are often seen as poor study designs to assess causality by themselves, but they can be very valuable in helping to develop ideas, as well as providing a “reality check” about the potential credibility of some hypothesis.^29^

Conversely, it is pointless to add yet another study, however perfect, showing what is already known very well – unless you have to do it for “political” purposes, say, for convincing decision makers in your own country.

Finally, it is not a good use of your time to chase something completely improbable or futile. For example, at the present state of the debate, it serves no purpose to add another study about the presence or absence of clinical benefits or harms of homeopathy: no one will change his or her mind about the issue.^30^,^31^ An exception might be something that is highly improbable, but that if true might lead to completely revolutionary insights – such an idea might be worth pursuing, even if the initial reaction of outsiders might remain incredulousness. Still, you should pursue unlikely hypotheses knowingly, that is, with the right amount of self-criticism – in particular, to make yourself aware when you are in a blind alley.

To keep yourself on the “straight and narrow”, it helps to form a group of people who cover different aspects of the problem you want to study: clinical, biochemical and physiological, and methodological – to discuss the project as equals. Such discussions can not only be tremendous fun but also will invariably lead to more profound and diverse research questions and will help to find solutions for practical as well as theoretical problems. In the right circumstances of a “machtsfreie Dialog”^32^ (a communication in which all are equal and that is only based on rational arguments and not on power – which all scientific debates should be), such a circle of colleagues and friends will help you to be self-critical.

Finally, when pursuing one’s research interests, one should be prepared to learn new skills from other fields or collaborate with others from these fields. If one stays only with the techniques and skills that one knows, it might not lead to the desired answers.^33^

### What if the data already exist? And you are employed to do a particular analysis with an existing protocol?

Even in the circumstance that the data already exist, it greatly helps to not jump into an analysis, but to think for yourself what you would ideally like to do – if there were no constraints. As Aldrich mentioned,^19^ also in that circumstance researchers should still

[…] begin their literature review and conceptual modeling as if they had the luxury of a blank slate […]. Writing without data constraints will, I believe, free their imaginations to range widely over the realm of possibilities, before they are brought to earth by practical necessities.

Moreover, this will make clear what compromises one will make by accepting the available data and the existing analysis protocol. Otherwise, one starts an analysis without being sufficiently aware of the limitations of a particular analysis on particular data.

### The difference between explanatory and pragmatic research

A useful distinction is between explanatory and pragmatic research: the former is research that aims at discovery and explanation, whereas the latter is intended to evaluate interventions or diagnostic procedures. The first type of research consists of chasing explanations by pursuing different and evolving hypotheses; the second type of research aims at making decisions about actions in future patients.^27^ The two opposites differ strongly in their thinking about the types of studies to pursue (eg, observational vs randomized), about the role of prior specification of a research hypothesis, about the need for “sticking to a prespecified protocol”, and about subgroup analyses and multiplicity of analyses. Some of these will be explained in the following subheadings.

The difference between explanatory and pragmatic trials is sometimes thought to mirror the difference between doing randomized trials versus observational research. However, even for randomized trials, a difference exists between “ pragmatic” and “explanatory” trials (coined first by Schwartz and Lellouch).^34^ Because it is not always easy to delineate what aspects of a randomized trial are “pragmatic” or “explanatory”, instruments have been crafted to help researchers and evaluators.^35^,^36^ Conversely, not all observational studies are explanatory: some are needed for pragmatic decisions (think about adverse effects of drugs and also about diagnostic evaluations where studies should influence practice guidelines) – while other studies aim at explaining how nature works.

### Which iterations should you allow yourself? Anticipating the next project

Thinking about a research problem is a strongly iterative process.^2^,^33^,^37^ One starts with a broad aim and then tries out several possible ideas about studies that might lead to better understanding or to better solutions.

Likewise, project proposals characteristically go through many iterations. In the early phases of the research, it is commonplace that the study design or even the research question is changed. Specific suggestions about common research problems and their potential solutions were given by Hulley and Cummings,^2^ which we reproduce in Appendix D.

The revision of the aims of a project may be profound, in particular in explanatory research (see “The difference between explanatory and pragmatic research” section), in contrast to pragmatic research (see “Shouldn’t you stick to a predefined protocol?” section). The chemist Whitesides wrote: “Often the objectives of a paper when it is finished are different from those used to justify starting the work. Much of good science is opportunistic and revisionist”.^38^ Along a similar line, Medawar proposed that to do justice to the real thought processes of a research undertaking, the discussion section of a paper should come at the beginning, since the thought processes of a scientist start with an expectation about particular results. The expectation determines which findings are of interest and why they will be interpreted in a particular way.^6^ He added that in real scientific life, scientists get new ideas (ie, new expectations) while doing their research, but “[…] many of them apparently are ashamed to admit, that hypotheses appear in their mind along uncharted byways of thought”.^6^

“Seeing something in the data” can be an important part of scientific discovery. This is often decried as “data dredging”, which it is not: one sees something because of one’s background knowledge and thereby there always is some “prior” that exists – even if that was not specified beforehand in the study protocol.^27^,^39^ The word “exploratory” is often misused when it is used to characterize a study. True “exploratory” data analysis would only exists if it is mindlessly done, such as a Genome Wide Association Study (GWAS) analysis – but even GWAS analyses have specific aims, which becomes clear when results are interpreted and some findings are designated as “important” and others not. As stated by Rothman:

Hypotheses are not generated by data; they are proposed by scientists. The process by which scientists use their imagination to create hypotheses has no formal methodology […]. Any study, whether considered exploratory or not, can serve to refute a hypothesis.^40^

Appendix Examples A5 and A7 show how projects changed mid-course because of a new discovery in the data or in the background knowledge about a research topic.

Generally, it is a good habit to think through what the next project might be, once you will have the result of the project you are currently thinking about, so as to know what direction your research might take.^33^

### Shouldn’t you stick to a predefined protocol?

Different research aims, in particular along the “explanatory” versus “pragmatic” continuum, may lead to different attitudes on the amount of change that protocols may endure while doing research.^27^,^39^ For randomized trials, and also for pragmatic observational research, the research question is usually fixed: does a new therapy lead to better outcomes for a particular group of patients in a particular setting? Because findings from randomized trials or pragmatic observational research may lead to millions of patients to adopt or avoid a particular therapy (which means that their well-being or even life depends on the research) researchers are generally not at liberty to change their hypotheses at the last moment – for example, by suddenly declaring an interest in a particular subgroup. They should stick to the predefined protocol. If a change is needed for practical reasons, it should be clearly stated in the resulting publications. This makes thinking about research questions and doing pilot studies beforehand all the more important (see “Pilot Study” section).

In contrast, much epidemiologic and clinical research tries to explain how nature works. This gives greater leeway: exploration of data can lead to new insights. Thus, “sticking to the protocol” is a good rule for randomized trials and pragmatic observational research, but may be counterproductive for explanatory research.^39^,^41^ Nevertheless, it is good to keep track of the changes in your thoughts and in the protocol, even if only for yourself. In practice, many situations are intermediate; in particular when using large available data sets, it often happens that one envisages in a protocol what one would do with the data, only to discover upon opening the data files that the data fall short or are more complex than imagined; this is another reason for doing pilot studies, even with large available data sets (see “Pilot Study” section).

### How much literature should you read?

If you are setting up a new research project in a new area, do not start by reading too much. You will quickly drown in the ideas of others. Rather, read a few general reviews that identify unanswered problems. Only return to the literature after you have defined your research question and provisionally your study design. Now, the literature suddenly becomes extremely interesting, since you know what types of papers you need. You also know what the potential objections and shortcomings are of the different design options, because you thought about them yourself. The number of relevant papers usually greatly shrinks, see Appendix Example A4.

### Shouldn’t you do a systematic review first?

It is argued that before embarking on a new piece of research, one should first do a systematic review and/or meta-analysis, because this may help to define the gaps in knowledge more precisely, and guide new research – or may show that the question has been solved. This argument is somewhat circular. A systematic review is a piece of research in itself, intended for publication, and requires much time and effort. Like any piece of research, it requires a clear research question. As such it does not “identify gaps”: a systematic review is about a research question which is already specified, but for which more information is needed. Thus, the main function of the advice to first do a systematic review is to know whether the research question that one has in mind has not yet been solved by others. Perusing the literature in depth is absolutely needed, for example, before embarking on a randomized trial or on a major observational study. However, this is not the same as doing a formal systematic review. In-depth scoping of the literature will suffice. If it is found that potentially valuable studies already exist on the research question that one has in mind, then the new study that one is thinking about may be discarded, and a systematic review should be done instead.

## Intermezzo: specific schemes to structure reasoning

Specific schemes have been proposed to guide our reasoning between the stage of delineation of the “gap in knowledge” and the stage of proposing the research design.

The acronym FINER (feasible, interesting, novel, ethical, and relevant) was coined by Hulley and Cummings^2^ and denotes the different aspects that one should consider to judge a budding research proposal. These words are a good checklist for an in-depth self-scrutiny of your research. The central aspects are the feasibility and whether the possible answers are exciting (and/or much needed).

The PICO format (Patient, Intervention, Control or Comparison, Outcome) is advocated by the evidence-based medicine and Cochrane movements and is very useful for clinical therapeutic research, particularly randomized controlled trials (RCTs).^1^,^42^ Questions about therapeutic interventions are highly specific, for example, a particular chemotherapeutic scheme (the intervention) is proposed to study survival (the outcome) among young women with a particular form of stage III breast cancer (the patients). This framework is less useful, and becomes a bit pointless, for etiologic research about generalizable questions such as: “Does smoking cause lung cancer?” which applies to all humans and to different types of smoking. Of course, all research will be done in particular population, with particular smoking habits, but this does not necessarily define the research question. Some of the first investigations about smoking and lung cancer were done in male doctors aged ≥35 years in the UK^43^ – this was a very convenient group to research, but being a male doctor in the UK is not part of the research question.

The PICO format is thus most applicable for pragmatic research. A much more detailed and elaborate scheme for pragmatic research was proposed by the US Patient-Centered Outcomes Research Institute (PCORI) which has published Methodology Standards, including “Standards for Formulating Research Questions”. While we would not agree with all six standards, junior investigators may find the structure useful as they think through their options – especially for pragmatic research questions.^44^

Counterfactual reasoning^3^ emphasizes those aspects of the “ideal randomized trial” that should be mimicked by an observational study. A key question is whether your study is addressing a hypothesis that could in theory be studied in a randomized trial. For example, if the research question is “does smoking cause lung cancer?”, then this is a question that could in theory (but not in practice) be addressed by randomizing study participants to be smokers or nonsmokers. In this situation, it may be useful to design your observational study with the intention of obtaining the same answer that would have been obtained if you had been able to do a randomized trial.

However, the aims of explanatory observational research are different from those of randomized trials.^27^ Explanatory research about disease etiology may involve “states” like being female, being old, being obese, having hypertension, having a high serum cholesterol, carrying the *BrCa1* gene, and so on, as causes of disease. None of these causes are interventions. In contrast, RCTs focus on what to do to change particular causes: which interventions are feasible and work? For example, being female might expose a person to job discrimination; the intervention might be to have women on the appointment committee or to use some kind of positive discrimination. Likewise, the gene for phenylketonuria leads to disease, but the intervention is to change the diet. For carriers of *BRCa1* genes, different strategies can be evaluated in RCTs to evaluate their effectiveness in preventing premature death due to breast cancer: frequent screening, prophylactic mastectomy, hormone treatment, and so on – which may have different effects. For obesity or hypertension or hypercholesterolemia, different types of interventions are possible – with potentially different effects and different adverse effects.

The interventionist outlook, that is, trying to mimic an RCT, can be very useful, for some type of observational studies, for example, about the adverse effects of drugs. It helps to make certain that one can mimic an “intervention” (ie, patients starting to use particular drugs) that is specific and consistent in groups of patients that are comparable (more technically, exchangeable – meaning that the results of the investigation would not change if the persons exposed and nonexposed were swapped). These conditions can be met in a credible way, if there are competing drugs for a similar indication, so that there is an active drug comparator: the interventions (use of different drugs in different patients) will be well defined, and the patients on the different drugs will tend to be comparable. This works particularly well if you are focusing on adverse drug effects that were unknown or unpredictable at the time of prescription.^45^,^46^ For example, you may obtain more valid findings in a study that compares the adverse effects of two different beta agonists for asthma care (ie, two different drugs within the same class), than to design a study which compares patients who are prescribed beta agonists with patients who are prescribed other asthma medication, or no medication at all – because the latter might be a highly different group of patients.^47^

As mentioned, there are some important studies about causes of diseases where a randomized trial is not feasible, even in theory. In particular, there are various “states” which are major causes of disease (obesity, cholesterol, hypertension, diabetes, etc). These states strongly affect the risks of disease and death, but cannot be randomized. For example, it is difficult to conceive of randomizing study participants to be obese or not obese; however, we could randomize them for the reduction of obesity, for example, through exercise, but such a study would assess the effects of a particular intervention, not of obesity itself. Still, it remains important to estimate the overall effects of obesity, that is, to answer the question “would this group of people have had different health status, on the average, if they had not been obese”. In this situation, the concept of “interventions” is not relevant to designing your study (at least in the way that the term “intervention” is commonly used). What is more relevant is simply to focus on the counterfactual contrast which is being assessed (eg, a body mass index [BMI] of 35 versus a BMI of 25), without specifying how this contrast came about.

A technique that has gone hand in hand with counterfactual reasoning in epidemiology is drawing DAGs; several introductions to DAG theory can be found in epidemiologic textbooks.^3^,^48^ DAGs can be useful in the brainstorming phase of a study, after the general research question has been defined. At this stage, a general structure for the study is envisaged and the complexity of the causal processes needs clarification. A DAG can be extremely useful for illustrating the context in which a causal question is being asked, the assumptions that will be involved in the analyses (eg, whether a particular risk factor is a confounder, a mediator, or a col-lider), and help us question the validity of our reasoning.^49^ Using DAGs helps us also decide which variables we need to collect information on and how they should be measured and defined. Given that DAGs root in causal thinking, their construction is, of necessity, subjective.

## Preparation: pilot study, protocol, and advance writing

### Doing a pilot study and collecting ancillary information about feasibility

May I now start? is a question heard after lengthy deliberations about the research question and the potential studies that follow from it. Such deliberations almost invariably produce a lot of enthusiasm and exhilaration – because they are fun. The researcher wants to begin collecting data or start the analysis. However, Crombie and Davies, in their chapter about “Developing the research question” state emphatically: “Don’t rush into a study”.^16^ Separate from doing a pilot study, which is about the procedures of your study, you may also need to collect ancillary information before actually starting your study.

#### Pilot study

Even if you think you are totally certain of what you want, you should first do a pilot study, based on a brief protocol.^2^,^22^ That initial protocol should be easy to write. You have already discussed the aim and design of your study. Write them down. You expect a particular type of information that is essential and that will tell the essence of your message (a particular 2-by-2 or X-by-Y table, a particular graph), which you can describe.

Pilot studies are not done to know the likely direction of the results; instead, the aim is to see whether you will be able to perform the procedures of your study – and ultimately whether that really is the study you want to do.^50^ The aim is to save yourself from embarrassment: data that very surprisingly do not turn out to be what you expected, questionnaires that are misunderstood or do not deliver the answers that you need or that are not returned, laboratories that do not produce, patients who do not show up, heads of other departments who block access to their patients or materials, or yourself who needs more time to manage the complexity of the undertaking.

We have never heard of someone who was sorry for having done a pilot. Conversely, we know many persons who found out at much personal embarrassment and institutional cost that their project was unfeasible. In intermediate cases, the pilot may show the need to change questionnaires or procedures before the study goes ahead.

In principle, a pilot study should be exactly like your final study and test out all your procedures on a small number of persons. Often, it is better to approach the task piecemeal and pilot different aspects of the research one by one.

A tough question is how to do pilot studies and pilot analyses when ethical or institutional review board approval is necessary for some of the actions in a pilot study. One solution might be to avoid piloting some procedures; for example, try parts of the procedure – for example, you may not be able to randomize in a pilot, but you may be able to try out data collection procedures and forms. There is a degree of circularity about piloting, also in obtaining funding, as one may need funding for the pilot. In practice, the best step might be to ask the ethics committee or review board of your institute which aspects of the research can be piloted and under what conditions.

In Appendix E, several questions that you might ask in pilot studies are listed. They may lead to profound reassessments of your research – particularly if you are piloting the collection of new data, but also if the research involves analyses of existing data.

#### Ancillary information

It may be necessary to collect additional information about event rates or standard deviations of measurements to calculate the statistical precision that might be obtained. Also, sometimes you need other ways of “testing the water” like procedures to streamlining data collection from different centers in order to know whether the study is feasible. Depending on the study size and importance, such activities may become studies in themselves and actually take a lot of time and money.

### Advance writing of paper: before full data collection and/or analysis

Whitesides’ advice is:

The key to efficient use of your and my time is that we start exchanging outlines and proposals as early in a project as possible. Do not, under any circumstances, wait until the collection of data is ‘complete’ before starting to write an outline.^38^

After the pilot study, you have a firm grasp of all elements that are necessary for a scientific paper: introduction, materials and methods, results, and discussion. In the introduction, you explain why you have done this research. Almost always, an introduction comprises three ideas: what is the general problem? what is the particular research question? what study will you perform to answer that question? This is followed by the materials and methods section. They have been extensively discussed and have been fine-tuned in the study protocol and the pilot study. Thereafter come the results sections. By now, you know what tables or figures you want and how you can obtain them, but not what the final numbers will look like. You will also have an idea about the auxiliary tables that you might need to explain your data to others (such as a table with the baseline characteristics or an additional table with a subgroup analysis). You can now draft the layouts of all these tables. Visualizing the presentation of your results in advance is the “bare minimum” of writing in advance.

Finally, the discussion section. Can you write a discussion before you know the final data? Of course you can; you even must think ahead. In principle, there are only three possible outcomes: the study can give the results that you hoped for; it can show the inverse; or something indeterminate in between. In all instances, you can imagine how you will react. One possibility is that you are disappointed by the results of your study, and you will tend to find excuses for why it did not produce the results you hoped for. What excuses might your produce? The other possibility is that it does show what you wanted; then you may have to imagine how others will react and what their objections might be. If the results are indeterminate, everybody might be disappointed, and you will need to explain the failure of your research to give clear-cut results. When you detect a specific weakness by imagining this situation, you may wish to change aspects of your study.

As we explain in Appendix F, there is no need to write a very extensive paper as a first draft – on the contrary, it might be more useful to write a short paper, which has the advantage that others will more readily read it and comment on it.

Never be afraid to discuss your study at all stages extensively with others, not only your immediate research colleagues but also semi-outsiders and also in this advance-writing stage. If you know, or are told by others, that a particular direction of your results might not be believed and therefore draw criticism because of some potential deficiency in your study, why not remedy it at this stage? Looking at what you have written, or by discussing potential results with others, you will be able to imagine more clearly what your readers and critical colleagues might object to.

Writing a paper beforehand is the ultimate test of whether the research project is what you wanted, whether your reasoning flows logically, or whether you forgot something. The initial draft will be a yardstick for yourself and for others – whatever happens during the course of your research. This will help you to surmount surprise happenings: you have written down where you started and why, and therefore you will also know very securely when and why you have to take a detour – or even a U-turn.

Writing is difficult and time-consuming. Writing a paper can easily take 5–10 revisions, which might span a full year (inclusive of the time it takes your supervisor or your colleagues to produce comments). During the writing, you will often be obliged to go back to the data and do additional or different analyses. Since your paper will need many revisions, and this will take such a long time, why not take a head-start at the beginning of your data collection? It will save frustration and lost time at the end of your project.

Many guidelines and advices exist about writing, both about the substance (how to use words and phrases) and about the process. All beginning researchers should have a look at some books and papers about writing, and seasoned researchers can still profit from rereading them. Several reporting guidelines exist for several types of studies (RCTs, observational, diagnostic research, etc). They are often very detailed, in describing what should be in title, abstract, and so on. Although they should not be mechanically adhered to,^28^ they help writing. In Appendix F, we have collected some wisdom that we particularly liked; several books on writing are listed, as well as reporting guidelines that help researchers to craft papers that are readable and contain all the information that is necessary and useful to others.

## Now you can start “your research”

After the piloting and after having written your paper, you are ready to start your data collection, your analysis, or whatever is needed to “do your research”.

The work that is needed before you can start to “do your research” will take a great deal of time and effort. What will you have achieved after setting up a piece of research following the lengthy and involved precepts of this paper? You will have specified a limited research question that you will solve. You will add one little shining stone to the large mosaic of science. At the time that you do the study, you may still be too close to see its effect on the overall picture. That will come over the years.

## Further reading

Some texts that we mention in the paper might be especially worthwhile for further reading; see Appendix G.
